# Oleic Acid Is not the Only Relevant Mono-Unsaturated Fatty Ester in Olive Oil

**DOI:** 10.3390/foods9040384

**Published:** 2020-03-26

**Authors:** Archimede Rotondo, Giovanna Loredana La Torre, Giacomo Dugo, Nicola Cicero, Antonello Santini, Andrea Salvo

**Affiliations:** 1 Department of Biomedical and Dental Sciences and Morpho-functional Imaging, University of Messina, Polo Universitario Annunziata, Viale Annunziata, 98168 Messina, Italy; llatorre@unime.it (G.L.L.T.); dugog@unime.it (G.D.); ncicero@unime.it (N.C.); 2 Department of Pharmacy, University of Napoli Federico II, via D. Montesano 49, 80131 Napoli, Italy; asantini@unina.it; 3 Department of Chemistry and Drug Technology, University of Roma La Sapienza, via P.le A. Moro 5, 00185 Roma, Italy; andrea.salvo@uniroma1.it

**Keywords:** *cis*-vaccenic, monounsaturated fatty, glycerols, NMR analysis, olive oil, *Capparis spinosa*, ^13^C-NMR, MARA-NMR

## Abstract

(1) Background: Extra-virgin olive oil (EVOO) is a precious and universally studied food matrix. Recently, the quantitative chemical composition was investigated by an innovative processing method for the nuclear magnetic resonance (NMR) experiments called Multi-Assignment Recovered Analysis (MARA)-NMR. (2) Methods: Any EVOO 13-carbon NMR (^13^C-NMR) profile displayed inconsistent signals. This mismatch was resolved by comparing NMR data to the official gas-chromatographic flame ionization detection (GC-FID) experiments: the analyses concerned many EVOOs but also the “exotic” *Capparis spinosa* oil (CSO). (3) Results: NMR and GC-FID evidenced the overwhelming presence of *cis*-vaccenic esters in the CSO and, more importantly, *cis*-vaccenic ^13^C-NMR resonances unequivocally matched the misunderstood ^13^C-NMR signals of EVOOs. The updated assignment revealed the unexpected relevant presence of *cis*-vaccenic ester (around 3%) in EVOOs; it was neglected, so far, because routine and official GC-FID profiles did not resolve oleic and *cis*-vaccenic signals leading to the total quantification of both monounsaturated fatty esters. (4) Conclusions: The rebuilt MARA-NMR and GC-FID interpretations consistently show a meaningful presence of *cis*-vaccenic esters in EVOOs, whose content could be a discrimination factor featuring specific cultivar or geographical origin. The study paves the way toward new quantification panels and scientific research concerning vegetable oils.

## 1. Introduction

Extra-virgin olive oil (EVOO) comes from the supernatant phase of juice obtained after cold pressing of *Olea europaea* fruits and is the fundamental dressing of any Mediterranean dish. It is considered the liquid gold in food trading because of its crucial role in the healthy way of life model called “Mediterranean Diet” [1]. Many scientific studies reveal that the chemical composition of EVOO is a perfect balance leading to countless benefits for humans [2,3,4,5]. The positive biological activities are reasonably due to the suitable presence of vegetable sterols [6], liposoluble polyphenols [7] and other anti-oxidant hydrocarbons [8] joined to the most abundant presence of mono-unsaturated tri-acyl-glycerol esters among the vegetable oils. Albeit the oleic ester in EVOOs is considered the overwhelming main mono-unsaturated fatty ester so far, this work casts another important mono-unsaturated fat potentially playing important biological roles. The wide impact of EVOOs composition accounts for the constantly updated European Regulation stating the chemical and taste features, limits and official analytical techniques recognized for olive oil trade [9,10]. In the last decades, the traditional food analysis was shocked by the nuclear magnetic resonance (NMR) as alternative quantitative (qNMR) approach [11] flanking the officially recognized separation techniques. The nondestructive NMR spectroscopy allows the *in-situ* detection of several chemical species without the requirement of a real physical separation [8,12,13]; moreover qNMR is feasible directly or through a clever data throughput [14,15]. The definite advantages of the NMR analyses are: a) minimal sample treatment [8], b) simultaneous detection of a great amount of data [16], c) reduction of systematic errors controlled by the intrinsic instrumental stability, d) constant and direct dependence between signal integration and quantitative values because of the constant nuclear magnetic momentum for the measured nuclei [17]. Criticism toward NMR concerned mainly sensitivity; however, it actually depends on the machine, on sample type, on used solvent, on observed nuclei and on specific experimental runs; this is the reason it should be evaluated from case to case [18]. After several years of research on EVOOs composition, Rotondo et al. have developed a Multi-Assignment Recovered Analysis (MARA-NMR) involving multi-nuclear ^1^H and {^1^H}^13^C-NMR experiments processed by an accustomed processing “MARA” algorithm [19]. This method successfully and quickly achieves the quantification of many components in EVOOs samples through high-resolution spectroscopy at 500 MHz (500 MHz HR-NMR). On the other hand, the “first” MARA-NMR scheme did not take into account some ^13^C-NMR resonances whose intensity was significant, but these were associated to EVOO minor components (theoretically negligible and contributing for less than 1%) and, for these studies, the best fitting goodness never reached the expected convergence. Since the official method for the quantitative determination of glyceryl fatty esters consists in the gas-chromatographic analysis of the corresponding methyl esters using the gas-chromatographic flame ionization detection (GC-FID) [20], this work focused on the data comparison between NMR and GC-FID on oils in order to solve inconsistent “leftovers” from literature. The paper evidences the neat importance of *cis*-vaccenic fatty ester in EVOOs as its content is around 3%; however, it was neglected so far because, according the official method, it is included in the level of oleic ester. 

## 2. Materials and Methods 

### 2.1. Materials and Samples

Deuterated chloroform with a small amount of Tetra-Methyl-Silane (TMS), used as internal reference, was purchased at reagent grade from Cambridge Isotope Laboratories (CIL) Inc. Extra-virgin olive oils were samples from awarded cultivars of different provenience representing top level food in the seasons 2014–2015. These samples were kindly given by producers in order to carry out scientific projects belonging to the BIOOIL program, aiming to improve knowledge about top quality products. 

Some seeds were isolated from *Capparis spinosa* fruits (known in Sicily as “cucunci”). Afterward seeds were dried in oven at 30 °C for 2 h. The matter was grinded in a mortar until the formation of a raw powder. This matter (20 g) was extracted in 100 mL of hexane, sonicated for 30 min at 30 °C and stirred overnight. The solution was then filtrated, and the hexane removed from the solution by using, at first, the rotating evaporator and later N_2_ flow over the sample. Finally, cucunci’s seed oil (CSO) was recovered (yield 15% *w*/*w*). 

### 2.2. GC-FID Analysis for the Comparative Tests

Fatty acids methyl esters (FAMEs) analysis was performed according to European Union (EU) Regulations [10]. It consists of the hydrolysis of tri-acyl-glycerides and cold transesterification with a methanol KOH solution; in particular, the methyl esters were prepared by vigorously shaking solution of the oil in heptane (0.1 g in 2 mL) with 0.2 mL of the methanolic KOH solution. The resulting solution was then injected into a gas chromatograph DANI MASTER GC-FID (Milan, Italy), equipped with a fused silica capillary column Phenomenex Zebron ZB-WAX (polar phase in polyethylene glycol) with a length of 30 m, internal diameter of 0.25 mm and film thickness of 0.25 µm. Helium was used as a carrier gas at a column flow rate of 1.2 mL/min, with a split ratio of 1:100. The temperature of the injector (split/splitless) and detector was of 220 °C and 240 °C, respectively. The oven was programmed as follows: initial temperature at 130 °C, final temperature at 200 °C (10 min) with an increase of 3 °C/min. The fatty acid methyl esters were identified by comparing the retention times with those of standard compounds. The relative percentage area of the fatty acids was obtained using the following relationship: %FAX = [AX/AT] × 100, where FAX stands for fatty acids to quantify, AX is the area of the methyl-esters and AT is the total area of the identified peaks in the chromatogram [21]. This analytical strategy is chosen for data comparison because it is officially recognized for the fatty esters quantification, on another hand the reader should be aware that the hydrolysis-esterification step is always tedious, laborious and time consuming, decreasing accuracy and precision. This is the reason why, lately, alternative analytical chromatographic methods have also been proposed [22], still showing limitations.

### 2.3. Sample Preparation for NMR

Sample preparation follows the same procedure successfully used by our group several years ago [7,8,12,19]. Briefly, all the CDCl_3_ solutions were kept homologous by mixing 122 μL of oil and 478 μL of deuterated chloroform (CDCl_3_) into a 5 mm test-tube (EVOO or CSO in a 13.5% weight ratio). In this study we used the same EVOOs studied in Reference 19; however, these were dissolved as different samples and the experiments were repeated in light of the new assignments. Tubes were immediately sealed to prevent solvent evaporation; it would affect the sample concentration influencing the chemical shift of many signals, especially the unsaturated and carbonyl ^13^C signals. These samples were readily used for the NMR scheduled analysis so that outcomes could be suitably processed and compared to each other. 

### 2.4. NMR Analysis

All the samples were analyzed at a constant temperature of 298 K on a 500 MHz Avance III NMR spectrometer endowed with a gradient assisted probe (SMARTprobe, Faellanden, Switzerland). The shimming procedure was carried out until the field homogeneity was assessed by less than 1.5 Hz of half-height line-width for the TMS signal. 

The 1D ^1^H and ^13^C{^1^H} NMR spectra were run at 499.74 and 125.73 MHz, respectively. This research exploited the analytical procedure including two experiments: a) the standard ^1^H experiment with 64 scans; b) the standard ^13^C NMR experiment with 32 scans. The entire procedure takes around 30 min of experimental time for any sample including preparation. Hard pulse for the maximum sensitivity (90° pulse), was calibrated and constantly checked for ^1^H throughout the samples being always 8.2 ± 0.1 μs at −6 dB. ^1^H-NMR experiments (type A) were run with a spectral width of 12 ppm, 64 scans, 10 s of acquisition time and 5 s of recycle delay in order to overcome problems coming from the differences in the proton relaxation times. For the same reason the ^13^C spectra (type B) were acquired with the 90° hard pulse (11.2 ± 0.3 us at 6 dB) with 32 scans, 5 s of acquisition time and 20 s for the time delay. Thanks to the MARA-NMR algorithm, these experimental elements were conveyed together for the overall quantitative evaluation.

### 2.5. NMR Processing and Data Treatment

All the spectra were processed through three main software programs (ACDLab/NMR 2012 (Toronto, Ontario, Canada), MestreNova 6.6.2 (Galicia, Spain), Topspin 4.0.5 (Bruker, Milan, Italy) and using several procedures for the coherent alignment, spectral phasing, calibration, base-line correction and integration procedure. The best processing choices are here reported regardless the many other adoptable procedures. Topspin processed data were selected with manual phase-correction, parametric base-line correction with an implemented polynomial curve (for example, for experiment I *absd 16* command). Calibration of experiment A was performed on the methyl group of the β-sitosterol signal to (δ_H_ = 0.738 ppm) with the TMS always being (δ = 0.0 ± 0.005 ppm); for ^13^C calibration of experiment B the divinyl- methylene group of the linoleate glycerols (L11; δ_13C_ = 25.6614 ppm) was used always keeping the known TMS ^13^C signal to δ_13C_ = 0.0 ± 0.05 ppm. The TMS calibration would not really change the results; here, the calibration over internal signals is preferred because these are less dependent on random conditions as explained elsewhere [19].

The serial integration of 100 regions for all the A-type experiments, and of 90 regions for experiment B profiles, provided a pretty big matrix whose columns were the 40 studied samples EVOO and rows represented 190 homologous integrations (see Supplementary Materials). Every column of this matrix was processed by the mentioned MARA algorithm [19]; this theoretical architecture is modified according to the original knowledge and assignments concerning *cis*-vaccenic esters (V). The experimental coherences simply confirm the presence of a relevant amount of V, also improving consistency assessed by low best fitting goodness (ρ) values. The extended procedure outputs up to 20 quantitative parameters [7,8,19] (Table 1) but this manuscript focuses on the 11 quantitative parameters showing sound precision and important significance (Table 2). The data validation and experimental error is evaluated through reproducibility (several samplings) and repeatability (analyses in different days of the same sample).

### 2.6. Mathematical Background of MARA-NMR and Updates

The used algorithm MARA-NMR was invented in this laboratory, exploiting the very simple idea that all NMR signals rise from active nuclei that belong to compounds and contribute according to: a) relative concentration, b) number of resonating nuclei, c) possible overlaps with homologous nuclei maybe belonging to other compounds [19]. If this theoretical statement and a suitable assignment is correct, the experimental profile should perfectly match our theoretical reconstruction. As explained in the original paper [18] experimental data are not ideal data-points, however we have designed this algorithm able to optimize quantitative parameters in order to minimize the overall deviations between experimental and theoretical outcomes enclosed in the function ρ which is the best-fitting goodness.

(1) $$\rho =\sum_{\mathrm{xj}=x1}^{\mathrm{xf}}\omega_{\mathrm{xj}}{\left(\frac{\gamma_{\mathrm{xj}}\;I_{\mathrm{xj}}}{I_{\mathrm{ref}}}-\frac{\sum_{i=a}^{n}N{}^{\circ}{\mathrm{NUC}}_{i}{*C}_{i}}{N{}^{\circ}{\mathrm{NUC}}_{\mathrm{ref}}{*C}_{\mathrm{ref}}}\right)}^{2}.$$

The intensity of any signal in the spectrum I_xj_ respect to a reference signal I_ref_ should even out the relative concentration (C_i_ against C_ref_) of the magnetically active nuclei NUC_i_ actually assigned to that signal. Coefficients ω and γ are empirical parameters able to reduce experimental deviation improving the algorithm; theoretically speaking the best fitting goodness ρ should be 0 but in the real world we accept low values. The introduction of 18 new assignments for the *cis*-vaccenic ester, by enhancing just one quantitative parameter referred to the “new” component greatly lowered the best fitting goodness giving the proof of concept about the assignment. The 20 quantitative parameters are derived by 11 expressions derived from A experiments and 65 expressions derived from B experiments put together in the same expression as equation (1) containing 76 *xj* members and 20 *i* compounds. In order to preserve the quantitative proportion of ^13^C integrations, despite the uneven nOe relayed on total decoupled carbon nuclei, adopted equations in the sum (1) are divided in blocks of nuclei with the same chemical environment (methyl terminal carbons, methylene inner chain carbons, vynil-methylene, etc.). It is demonstrated that MARA-NMR keeps the quantitative information as reported in Supplementary Materials and in Reference [19].

## 3. Results

**Figure 1:**
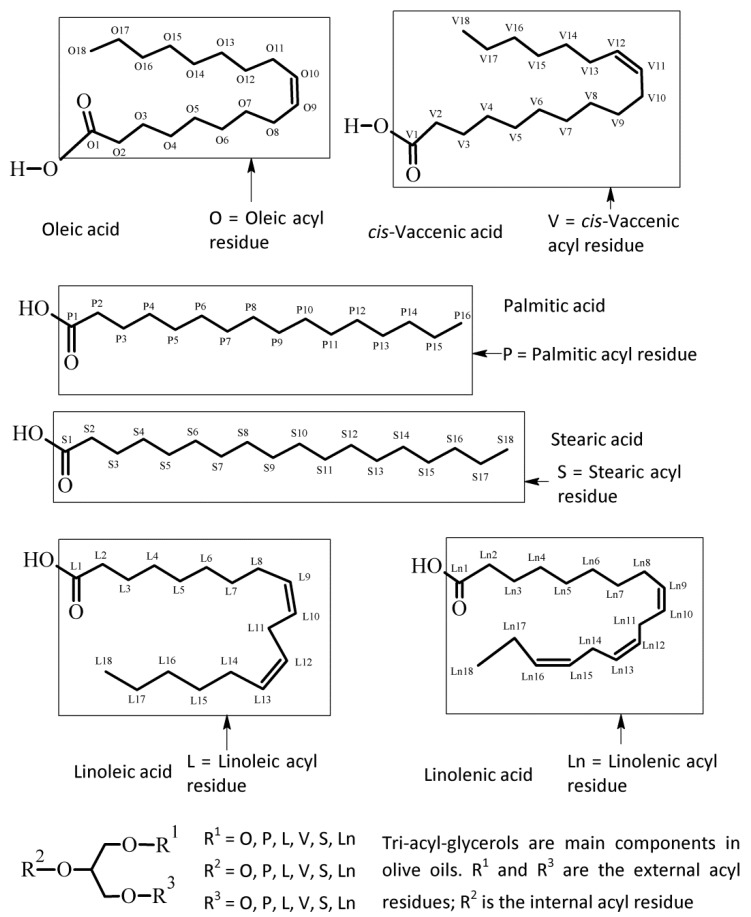
Chemical scheme of the fatty esters commonly found in olive oils with relative abbreviation. Usually these acyl residues are esters of the glycerol moiety. The labelling scheme of carbon atoms is adopted in this paper for assignments and discussion.

**Figure 2:**
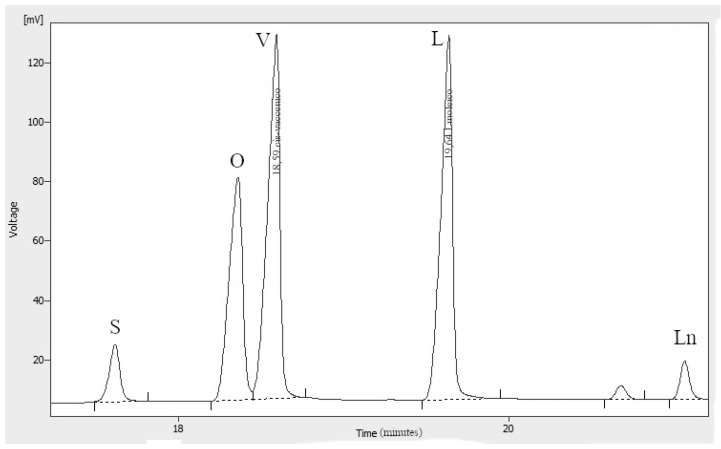
Expanded region of interest in the gas-chromatographic flame ionization detection (GC-FID) profile for *Capparis spinosa* oil; oleic (O) and *cis*-vaccenic (V) methyl esters are resolved for the quantification. In the case of extra-virgin oil the O peak is around 20 times more than V. Other labelled signals are linolenic (Ln), linoleic (L) and stearic (S) esters

**Figure 3:**
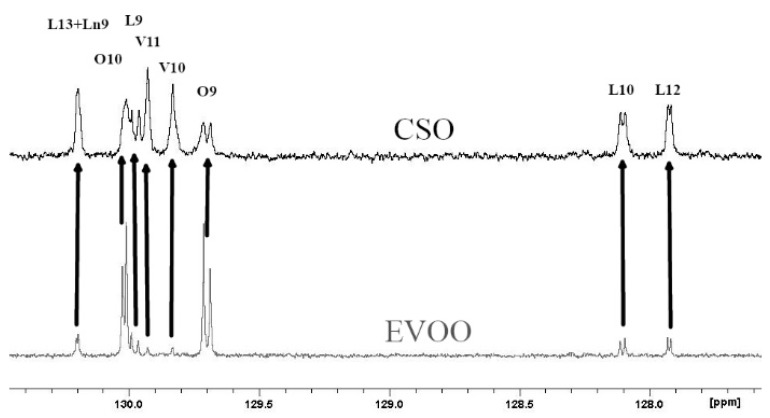
^13^C-NMR profiles for olive oil (EVOO) in gray and capparis seed oil (CSO) in black. All the assignments for oleic (O), linoleic (L) linolenic (Ln) and *cis*-vaccenic (V), with the number representing carbon atom position respect to the 1 carboxyl position, are pretty known and coherent with quantitative and literature data.

Figure 1 shows the chemical moieties, related abbreviations and the adopted labelling scheme; Figure 2 reports the GC-FID profile referring to the CSO extracted in our laboratory and Figure 3 represents the ^13^C-NMR profile of EVOO and CSO in the unsaturated region (127–131 ppm) along with the relative assignment witnessing the presence of the *cis*-vaccenic ester. As easily foreseeable, other NMR spectral regions also clearly showed *cis*-vaccenic resonances; however, a total assignment of 18 ^13^C carbon atoms was challenged by the many overlaps. Previous pioneering studies pointed out the challenging quantitative decoding of the mono-unsaturated fatty esters mixture in EVOOs [23]. Specifically, other minor mono-unsaturated fatty esters (MUFE) were taken into account; beyond the oleic (O) are also considered cis-vaccenic (V), eicosenoic (E) and palmitoleic (PO) [24,25]. On the other hand, data coming from known EVOOs compositions, limit the quantitative contribution of E and PO below 1% [9] and it is consistently witnessed by the lack of defined resonances in the regions where these esters should not have overlap with other similar constructs. The Multiple Assignment Recovered Analysis (MARA-NMR) takes advantage of any spectral section also overcoming the overlap issues hampering, so far, the independent quantification of mono-unsaturated fatty esters. Specifically, in this case, MARA-NMR processing definitely led to the detection and quantification of the V esters (consistently all over the recorded spectral span). Among the 20 variables feed out from MARA-NMR whose code is reported in Table 1, we herein have restricted our considerations to the most meaningful 11 variables reported in Table 2 along with the relative standard deviation. 

**Figure 4:**
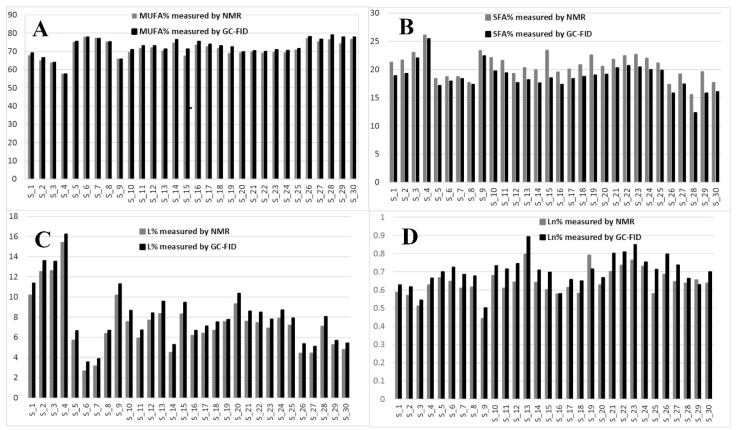
Comparison between MARA-NMR and GC-FID measured quantitative parameters referred to: (**A**) mono-unsaturated (MUFA), (**B**) saturated (SFA), (**C**) Linoleic (L) and (**D**) Linolenic (Ln) esters in relative percent ratio.

With respect to the other studies [24,25] the new information remarkably smooths discrepancies between ^1^H and ^13^C-NMR as the mono-unsaturated fatty esters contribution in ^1^H-NMR matches the contribution of O and V esters, which actually should be also somewhat enhanced by the minor PO and E esters’ contribution. Because of the tricky GC-FID resolution between V and O, also referred to in the European regulation (which suggests to report the whole V+O contribution), it is not always possible to compare GC and NMR data. However, the new available data, display the best fitting so far obtainable (Figure 4) concerning the measurements of mono-unsaturated (O + V + PO), saturated (P + S), di-unsaturated (L) and tri-unsaturated (Ln) fatty esters. The average V contribution is around 3% and it is consistent with previous NMR [23] and GC-FID [26] analyses; on the other hand, we think that MARA-NMR is the most versatile method suitable for serial processing of several samples and data. We think that this remarkable parameter in EVOOs cannot be ignored, since it is not constant by shifting from sample to sample, therefore it could assess specific features of different food products. The V quantification is not a marker for this study according to Table 2; however, it will trigger many important statistical considerations. 

This enlightened an important piece of information concerning the *cis*-vaccenic ester as main compound in CSO but also as relevant ester contributing to the EVOO mixture. This last element was incredibly ignored so far. Table S1 (Supplementary Materials) reports the extended panel of 20 quantitative variables considered in the study for 33 samples (see details in Supplementary Materials). These values are obtained by MARA-NMR—a post-processing algorithm working over the two experiments A and B type.

## 4. Discussion

The previously reported assignments for EVOOs ^13^C-NMR definitely accounted for five fatty esters in the following quantitative order: oleic (O), palmitic (P), linoleic (L), stearic (S) and linolenic (Ln) [27,28]. Some other tentative assignments concerned mono-unsaturated fatty esters like palmitoleic (PO) and 11-eicosenoic (E) constructs [29]. Despite the wide availability of NMR reports [30], none of these clearly explained the systematic presence of unknown resonances (in our processing batch at 129.92, 129.82, 31.82, 22.69 ppm and others) which account for a relevant quantitative contribution (around 3%, Figure 3). Scientific hesitancy probably owes to the general opinion that the total amount of other fatty esters is limited to less than 1% of EVOOs. This idea was questioning the NMR technique itself as possible analytical method but the serendipitous extraction of the *Capparis spinosa* oil (CSO) allowed us to solve this inconsistency because of the remarkable presence of *cis*-vaccenic (V) esters. The comparison between NMR and GC-FID analyses of CSO consistently confirmed the main presence of the V ester with a minor contribution of the O ester. The analogous analytical approach executed over several EVOO samples made us realize that the detected mono-unsaturated fatty esters were again O and V but in a reversed quantitative proportion respect to the CSO. Against this background, the main NMR resonances attributed to V in other peculiar food matter [31,32,33] (as also the reported CSO sample) were matching the EVOO signals as reported in Figure 3. It definitely gave us the chance to include the V component in the EVOO quantitative panel according to the ^13^C-NMR resonances afore mentioned. The V remarkable presence is not just a production side product as we did not observe the presence of *trans* isomers (resonances downfield respect 5.40 ppm in the ^1^H-NMR and relative other singlets in the ^13^C-NMR). Once again these results confirm the stability and sound presence of the *cis* form of unsaturated esters in spite of the minor thermodynamic stability. In order to perform the updated comprehensive quantitative NMR analysis of EVOO we have adopted an accustomed procedure based on MARA-NMR. Although it is not the first analytical comparison between GC and NMR [34,35], the novel MARA-NMR strategy suitably refined according to the new information led to a very good fitting (Figure 4). The whole outcome is reported in Tables (Table 1 and Table 2, and Tables S1 and S2 in Supplementary Data). In order to get consistent data, we have chosen to compare the percent presence of L and Ln as detected, whereas the saturated fatty esters (SFA%) were considered as the sum S+P and the mono-unsaturated fatty esters (MUFA) were considered as O+V+PO. We think it is actually an important parallel evaluation whose general trend shows a very good fitting also kept with samples showing sensibly different proportions. Finally, by properly considering all the mono-unsaturated fatty esters, the MUFA% estimation reached an unprecedented very good matching. On the other hand, the slight systematic overestimation of GC-FID respect to the NMR for L% and Ln% and underestimation of SFA% deserves to be elucidated with further studies requiring standard mixtures similar to EVOO, which is a tri-acyl-glycerol mixture. At the moment, these substrates are not available but work is in progress to develop further information. Although it is not the first case of V detection and also quantification [36], the EVOOs routine quantifications barely evidence the resolution for O-V peaks; this is clearly shown in the GC picture of the European Regulation 2013 [9]. Our observations also demonstrated that new GC-FID columns keep a better (affordable) resolution, whereas routine instruments adopted for serial records easily present the V peak as O shoulder. Fortunately, recorded ^13^C-NMR provide the missing information about the V fraction (not really taken into account so far) for any EVOO sample (Figure 3). According to our opinion, future studies could take advantage from a “powered” MARA-NMR working over sensitivity-enhanced ^13^C-NMR profile (optimized scans); these could push further the frontiers of quick qNMR in EVOOs by enabling the independent quantification of fatty esters in the 2- internal position of glycerides but also the improved quantification of other minor components (see Supplementary Materials). This contribution also opens the way toward new studies concerning sensory attributes, geographical origin and beneficial effects [37] of EVOO as fundamental functional food with the major presence of glycerol esters [38]. 

## 5. Conclusions

This study definitely assesses the constant and relevant presence in olive oils of a not-oleic mono-unsaturated fatty ester called *cis*-vaccenic ester. It resolves the literature controversies concerning the assignment of some ^13^C-NMR resonances but, more importantly, it brings back the expected coherency between NMR and chromatography data. The serendipitous comparison of GC-FID and NMR profiles for the “exotic” *Capparis spinosa* oil evidenced the overwhelming amount of *cis*-vaccenic ester in this matrix but also unambiguously confirmed ^13^C-NMR assignments also validated in olive oil. By reconsidering the NMR and GC-FID of olive oils, it turned out the surprising quantitative contribution (around 3%) of *cis*-vaccenic ester. The official GC method does not always perform the required resolution to resolve and quantify oleic and *cis*-vaccenic esters and this is leading to the undistinguished quantification of both mono-unsaturated fatty esters. It opens up great potential for any technique able to clearly resolve *cis*-vaccenic moieties (just like ^13^C-NMR) in the study of extra-virgin olive oils. 

## Figures and Tables

**Table 1:** Abbreviations used to indicate quantitative values.

tri-acyl-glycerol percent	TG%
1,2 di-acyl-glycerol percent	1,2-DG%
1,3 di-acyl-glycerol percent	1,3-DG%
squalene molecular%	SQ_mol_%
linolenate esters %	Ln%
linoleates esters %	L%
oleic esters %	O%
palmitoleic esters %	PO%
*cis*-vaccenic esters %	V%
palmitate esters %	P%
stearate esters	S%
linolenate esters % in internal glyceril position	Lni%
linoleates esters % in internal glyceril position	Li%
oleic esters % in internal glyceril position	Oi%
palmitoleic esters % in internal glyceril position	POi%
*cis*-vaccenic esters % in internal glyceril position	Vi%
palmitate esters % in internal glyceril position	Pi%
sterarate esters % in internal glyceril position	Si%
β-sitosterol + avenasterol + camposterol in molecular ppm	VSTR
cyclo arthenol and other cyclosterols in molecular ppm	CYSR

**Table 2:** Quantitative data and relative deviation for 11 main variables (whose code is reported in Table 1), as measured through Multi-Assignment Recovered Analysis-Nuclear Magnetic Resonance (MARA-NMR) processing method working on mono dimensional ^1^H and ^13^C-NMR experiments for 33 samples. Standard deviations were measured through 9 different experiments on 3 identical samples analyzed on three different days.

Sample	TG%	1,2-DG%	1,3-DG%	SQ_mol_%	Ln%	L%	O%	PO%	V%	P%	S%
S_1	96.7 ± 0.1	1.19 ± 0.06	2.1 ± 0.1	1.7 ± 0.1	0.59 ± 0.03	10.2 ± 0.1	63.9 ± 0.5	1.3 ± 0.3	2.8 ± 0.3	19.1 ± 0.3	2.2 ± 0.2
S_2	97.4 ± 0.1	1.30 ± 0.07	1.34 ± 0.09	2.2 ± 0.1	0.57 ± 0.03	12.5 ± 0.1	61.8 ± 0.4	0.9 ± 0.2	2.5 ± 0.2	19.7 ± 0.3	1.9 ± 0.2
S_3	97.7 ± 0.1	1.65 ± 0.09	0.68 ± 0.05	1.3 ± 0.1	0.51 ± 0.03	12.6 ± 0.1	59.4 ± 0.4	0.8 ± 0.2	3.7 ± 0.3	21.1 ± 0.3	2.0 ± 0.2
S_4	97.4 ± 0.1	1.39 ± 0.07	1.21 ± 0.08	0.8 ± 0.0	0.63 ± 0.03	15.4 ± 0.1	51.9 ± 0.4	1.1 ± 0.2	4.9 ± 0.4	23.7 ± 0.3	2.4 ± 0.2
S_5	97.2 ± 0.1	2.1 ± 0.1	0.70 ± 0.05	2.9 ± 0.2	0.67 ± 0.04	5.7 ± 0.1	72.8 ± 0.5	0.7 ± 0.1	1.8 ± 0.2	16.0 ± 0.2	2.4 ± 0.2
S_6	97.3 ± 0.1	1.70 ± 0.09	0.97 ± 0.07	2.7 ± 0.2	0.65 ± 0.03	2.6 ± 0.0	73.9 ± 0.5	0.7 ± 0.1	3.5 ± 0.3	16.7 ± 0.2	1.9 ± 0.2
S_7	97.3 ± 0.1	2.0 ± 0.1	0.77 ± 0.05	2.5 ± 0.2	0.61 ± 0.03	3.1 ± 0.0	74.2 ± 0.5	0.8 ± 0.2	2.7 ± 0.2	16.1 ± 0.2	2.5 ± 0.2
S_8	97.7 ± 0.1	1.59 ± 0.08	0.74 ± 0.05	2.2 ± 0.1	0.61 ± 0.03	6.4 ± 0.1	70.8 ± 0.5	0.9 ± 0.2	3.6 ± 0.3	15.5 ± 0.2	2.1 ± 0.2
S_9	96.8 ± 0.1	1.78 ± 0.09	1.4 ± 0.1	1.3 ± 0.1	0.44 ± 0.02	10.2 ± 0.1	60.0 ± 0.4	1.1 ± 0.2	5.0 ± 0.4	21.7 ± 0.3	1.6 ± 0.1
S_10	96.9 ± 0.1	1.30 ± 0.07	1.8 ± 0.1	2.3 ± 0.1	0.68 ± 0.04	7.6 ± 0.1	65.2 ± 0.5	1.1 ± 0.2	3.4 ± 0.3	20.0 ± 0.3	2.1 ± 0.2
S_11	97.4 ± 0.1	1.21 ± 0.06	1.4 ± 0.1	2.9 ± 0.2	0.61 ± 0.03	5.9 ± 0.1	67.2 ± 0.5	1.2 ± 0.3	3.6 ± 0.3	19.2 ± 0.3	2.4 ± 0.2
S_12	97.6 ± 0.1	1.32 ± 0.07	1.04 ± 0.07	3.1 ± 0.2	0.64 ± 0.03	7.7 ± 0.1	65.4 ± 0.5	2.5 ± 0.5	4.6 ± 0.4	17.4 ± 0.3	1.9 ± 0.2
S_13	97.7 ± 0.1	1.47 ± 0.08	0.88 ± 0.06	2.8 ± 0.2	0.79 ± 0.04	8.3 ± 0.1	67.1 ± 0.5	1.0 ± 0.2	2.4 ± 0.2	18.3 ± 0.3	2.0 ± 0.2
S_14	97.8 ± 0.1	1.40 ± 0.07	0.80 ± 0.06	3.4 ± 0.2	0.64 ± 0.03	4.5 ± 0.0	71.3 ± 0.5	1.2 ± 0.2	2.5 ± 0.2	17.7 ± 0.3	2.3 ± 0.2
S_15	97.8 ± 0.1	1.14 ± 0.06	1.07 ± 0.07	4.0 ± 0.3	0.60 ± 0.03	8.3 ± 0.1	64.0 ± 0.5	0.7 ± 0.1	3.1 ± 0.3	20.7 ± 0.3	2.7 ± 0.2
S_16	97.8 ± 0.1	1.48 ± 0.08	0.72 ± 0.05	3.1 ± 0.2	0.58 ± 0.03	6.2 ± 0.1	70.2 ± 0.5	0.6 ± 0.1	3.0 ± 0.3	17.5 ± 0.3	2.0 ± 0.2
S_17	97.4 ± 0.1	1.53 ± 0.08	1.08 ± 0.07	2.1 ± 0.1	0.61 ± 0.03	6.4 ± 0.1	68.4 ± 0.5	1.2 ± 0.3	3.3 ± 0.3	17.9 ± 0.3	2.2 ± 0.2
S_18	97.4 ± 0.1	1.22 ± 0.06	1.4 ± 0.1	2.3 ± 0.1	0.58 ± 0.03	6.7 ± 0.1	68.5 ± 0.5	0.8 ± 0.2	2.5 ± 0.2	19.1 ± 0.3	1.7 ± 0.1
S_19	97.6 ± 0.1	1.43 ± 0.07	0.98 ± 0.07	3.5 ± 0.2	0.79 ± 0.04	7.5 ± 0.1	65.6 ± 0.5	0.7 ± 0.2	2.8 ± 0.3	20.7 ± 0.3	1.9 ± 0.2
S_20	97.2 ± 0.1	1.59 ± 0.08	1.17 ± 0.08	2.0 ± 0.1	0.63 ± 0.03	9.3 ± 0.1	64.8 ± 0.5	1.1 ± 0.2	3.7 ± 0.3	19.0 ± 0.3	1.5 ± 0.1
S_21	97.2 ± 0.1	1.70 ± 0.09	1.10 ± 0.08	3.0 ± 0.2	0.70 ± 0.04	7.6 ± 0.1	66.2 ± 0.5	0.6 ± 0.1	3.1 ± 0.3	19.9 ± 0.3	1.9 ± 0.2
S_22	97.7 ± 0.1	1.49 ± 0.08	0.78 ± 0.05	3.6 ± 0.2	0.73 ± 0.04	7.5 ± 0.1	64.8 ± 0.5	1.3 ± 0.3	3.4 ± 0.3	20.7 ± 0.3	1.7 ± 0.1
S_23	98.0 ± 0.1	1.63 ± 0.09	0.37 ± 0.03	3.4 ± 0.2	0.76 ± 0.04	6.9 ± 0.1	65.4 ± 0.5	1.4 ± 0.3	2.8 ± 0.3	20.1 ± 0.3	2.5 ± 0.2
S_24	97.6 ± 0.1	1.58 ± 0.08	0.85 ± 0.06	3.4 ± 0.2	0.73 ± 0.04	7.9 ± 0.1	65.9 ± 0.5	1.1 ± 0.2	2.5 ± 0.2	20.0 ± 0.3	2.0 ± 0.2
S_25	98.0 ± 0.1	1.21 ± 0.06	0.78 ± 0.05	2.4 ± 0.2	0.58 ± 0.03	7.2 ± 0.1	66.9 ± 0.5	0.6 ± 0.1	3.6 ± 0.3	19.2 ± 0.3	2.0 ± 0.2
S_26	97.3 ± 0.1	1.27 ± 0.07	1.4 ± 0.1	2.0 ± 0.1	0.68 ± 0.04	4.4 ± 0.0	73.3 ± 0.5	1.3 ± 0.3	3.0 ± 0.3	15.6 ± 0.2	1.8 ± 0.1
S_27	97.4 ± 0.1	1.48 ± 0.08	1.13 ± 0.08	2.5 ± 0.2	0.64 ± 0.03	4.4 ± 0.0	72.2 ± 0.5	0.9 ± 0.2	2.6 ± 0.2	16.9 ± 0.2	2.3 ± 0.2
S_28	97.8 ± 0.1	1.31 ± 0.07	0.85 ± 0.06	2.5 ± 0.2	0.64 ± 0.03	7.1 ± 0.1	73.9 ± 0.5	1.3 ± 0.3	1.5 ± 0.1	13.7 ± 0.2	1.8 ± 0.1
S_29	96.9 ± 0.1	1.45 ± 0.08	1.6 ± 0.1	2.7 ± 0.2	0.65 ± 0.04	5.2 ± 0.0	70.5 ± 0.5	1.4 ± 0.3	2.6 ± 0.2	17.1 ± 0.2	2.4 ± 0.2
S_30	97.9 ± 0.1	1.22 ± 0.06	0.89 ± 0.06	1.9 ± 0.1	0.64 ± 0.03	4.8 ± 0.0	73.1 ± 0.5	0.9 ± 0.2	2.9 ± 0.3	15.4 ± 0.2	2.3 ± 0.2
S_31	96.9 ± 0.1	2.5 ± 0.1	0.50 ± 0.03	2.6 ± 0.2	0.56 ± 0.03	6.3 ± 0.1	64.5 ± 0.5	0.5 ± 0.1	5.2 ± 0.5	21.5 ± 0.3	1.4 ± 0.1
S_32	96.0 ± 0.1	3.0 ± 0.2	0.99 ± 0.07	3.1 ± 0.2	0.69 ± 0.04	9.4 ± 0.1	61.8 ± 0.4	1.0 ± 0.2	4.4 ± 0.4	21.1 ± 0.3	1.8 ± 0.1
S_33	95.8 ± 0.1	2.3 ± 0.1	1.9 ± 0.1	2.2 ± 0.1	0.66 ± 0.04	8.7 ± 0.1	65.6 ± 0.5	1.0 ± 0.2	3.9 ± 0.4	18.3 ± 0.3	1.9 ± 0.1

## Supplementary Materials

The following are available online at https://www.mdpi.com/2304-8158/9/4/384/s1, Table S1: Analyzed samples coming from awarded BIOOIL competition 2014. The used code is connected to the provenance and to the known cultivar. Table S2: Quantitative data and relative deviation for 20 main variables, as measured through MARA-NMR processing method working on mono dimensional ^1^H and ^13^C-NMR experiments for 33 samples. Table S3: General scheme of MARA-NMR referred just to the first sample. There are several blocks: namely ^1^H-NMR integrations with assignment (100 entries), DPFGSE ^1^H-NMR integrations (17 entries, not used in this study), ^13^C-NMR integrations (90 entries) along with some sum of integrals belonging to the same spectral block. Where possible, assignments are performed respecting the chemical position indicated also in other studies about the NMR of olive oil compounds (see Figure 1), for the fatty esters the abbreviation is followed by a number indicating the distance from the carboxyl position (generally from 1 to 18). These first rows will be used in the following equations according to the style of (1) (see main text) conveyed as square sum in the raw called RHO. The RHO value is minimized playing around with the quantitative variables so that the theoretical outcome is best-fitting the real (independent) variables, Figure S1. Stack-plot of eight olive oils coming from Sicily. The reported assignment follows the labeling used in the main manuscript (scheme 1). The expanded regions around 22 and 32 ppm show the clear presence of cis-vaccenic acid signals useful for the quantification within MARA-NMR quantification. The aromatic region is already reported in Figure 2 of the main text.
