# Supplementary material for: Oleic Acid Is not the Only Relevant Mono-Unsaturated Fatty Ester in Olive Oil
Source: Foods. 2020 Mar 26;9(4):384. doi: 10.3390/foods9040384 (PMC7230673; doi:10.3390/foods9040384)
Supplement: Supplementary file 1 [file foods-09-00384-s001.pdf]

foods

MDPI

Article Supplementary Material

Oleic Acid is not the Only Relevant Mono-Unsaturated Fatty Ester in Olive Oil

Archimede Rotondo [1,*], Giovanna Loredana La Torre [1], Giacomo Dugo [1], Nicola Cicero [1], Antonello Santini [2] and Andrea Salvo [3]

[1] Department of Biomedical and Dental Sciences and Morpho-functional Imaging, University of Messina, Polo Universitario Annunziata, Viale Annunziata 98168 Messina, Italy; llatorre@unime.it (G.L.L.T.); dugog@unime.it (G.D.); ncicero@unime.it (N.C.)
[2] Department of Pharmacy, University of Napoli Federico II, via D. Montesano 49, 80131 Napoli, Italy; asantini@unina.it
[3] Department of Chemistry and Drug Technology, University of Roma La Sapienza, via P.le A. Moro 5, 00185 Roma, Italy; andrea.salvo@uniroma1.it
* Correspondence: arotondo@unime.it; Tel.: 39-090-676-6890

Received: 26 February 2020; Accepted: 19 March 2020; Published: date

Abstract: (1) Background: Extra-virgin olive oil (EVOO) is a precious and universally studied food matrix. The quantitative chemical composition was interpreted by an innovative processing method for the nuclear magnetic resonance (NMR) experiments called MARA-NMR. Nonetheless, any EVOO 13-carbon NMR (^{13}C-NMR) profile displayed inconsistent signals. (2) Methods: This inconsistency was resolved by NMR data comparison to the official gas-chromatographic (GC-FID) experiments: these analyses concerned many EVOOs but also the “exotic” Capparis Spinosa Oil (CSO). (3) Results: NMR and GC-FID consistently evidenced the overwhelming presence of cis-vaccenic esters in the CSO and, more importantly, cis-vaccenic ^{13}C-NMR resonances unequivocally matched the misunderstood ^{13}C-NMR signals of EVOOs. The updated assignment revealed the unexpected relevant presence of cis-vaccenic ester (around 3%) in EVOOs; it was neglected, so far, because routine and official GC-FID profiles did not resolve oleic and cis-vaccenic signals leading to the total quantification of both mono unsaturated fatty esters. (4) Conclusions: The rebuilt MARA-NMR and GC-FID interpretations consistently show a meaningful presence of cis-vaccenic in EVOOs, whose content is a potential discrimination factor featuring specific cultivar or geographical origin. The study paves the way toward new quantification panels and scientific research concerning vegetable oils.

Keywords: cis-vaccenic; mono-unsatured fatty; glycerols; NMR analysis; olive oil; Capparis Spinosa; ^{13}C-NMR; MARA-NMR.

1. Introduction

Supplementary file reports extended tables available and extracted through MARA-NMR procedure; just some of the quantitative entries with good deviation and significance are used in the main text, whereas here we also present other data which potentially would boost the application of NMR for the EVOO analysis. The main future target is the quantification of the occurrence of fatty esters in specific glyceridic position (these could be present in the external 1,3- or internal 2-position) but also the determination of sterols, polyphenols and so on.

2. Results

2.1. Figures

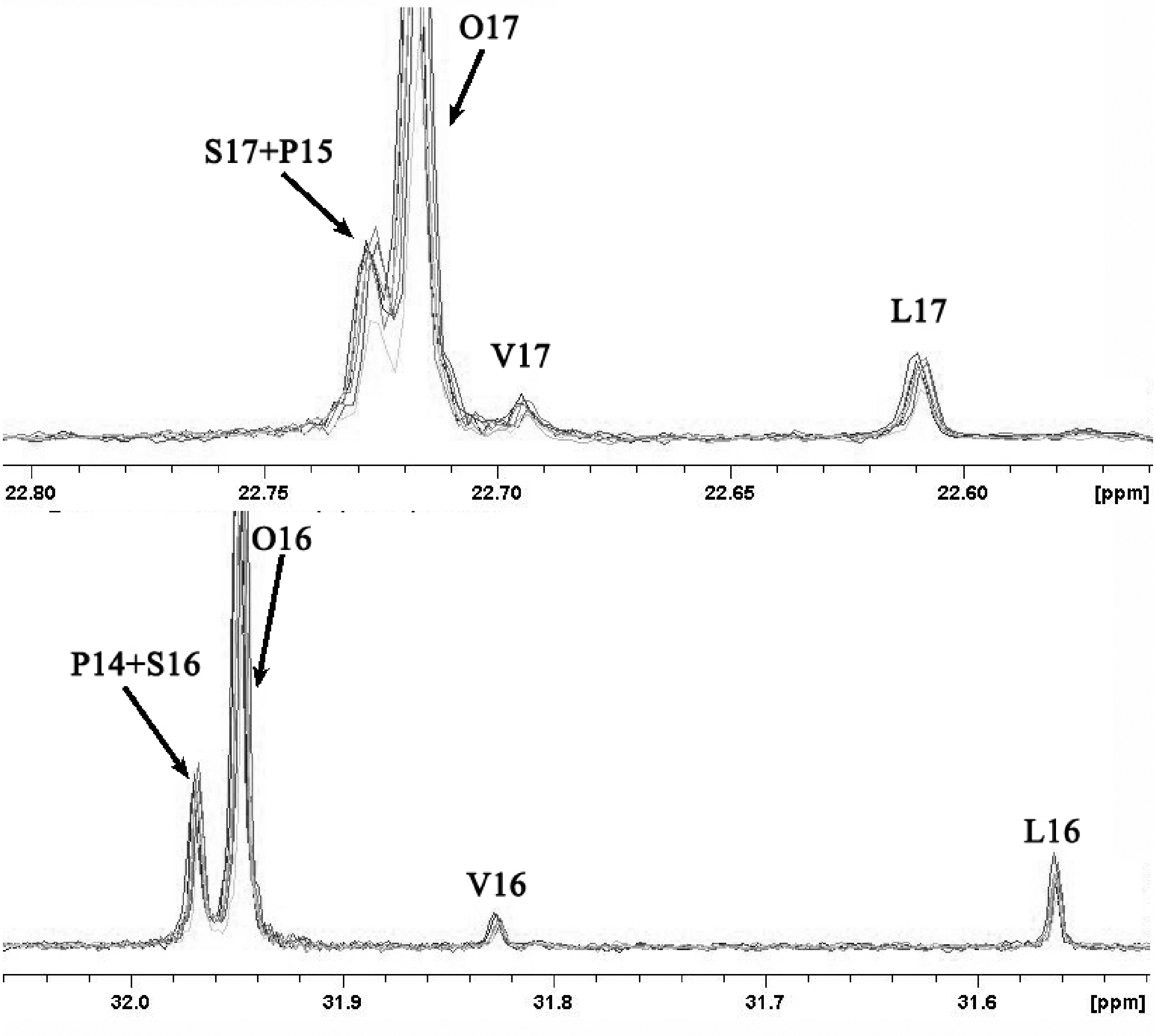

Figure S1. Stack-plot of eight olive oils coming from Sicily. The reported assignment follows the labeling used in the main manuscript (scheme 1). The expanded regions around 22 and 32ppm show the clear presence of cis-vaccenic acid signals useful for the quantification within MARA-NMR quantification. The aromatic region is already reported in figure 2 of the main text.

2.2. Tables

Tables are extended explanation of the sample type and provenance with also detailed quantitative results from MARA-NMR scheme.

Table S1. Analysed samples coming from aworded BIOOIL competition 2014. The used code is connected to the provenance and to the known cultivar.

Used Code	Lab. Code	Provenience	EVOO Specific Information
S_1	TUR_5	Turkey	Adremittion monovarietal
S_2	TUR_6	Turkey	Domat monovarietal
S_3	TUN_7	Tunisia	blend
S_4	TUN_8	Tunisia	Chehiall
S_5	SPA_17	Spain	Hojiablanca monovarietal

S_6	SPA_18	Spain	Hojiablanca monovarietal
S_7	SPA_20	Spain	Hojiablanca monovarietal
S_8	SPA_22	Spain	Picual monovarietal
S_9	SPA_23	Spain	Arbequina
S_10	PGL_27	Portugal	Calega Vulgar monovarietal
S_11	PGL_28	Portugal	Cobrancosa Frantoio
S_12	PGL_29	Portugal	Verderal Madural blend
S_13	PGL_30	Portugal	blend
S_14	PGL_31	Portugal	blend
S_15	CAL_40	Calabria (Italy)	Cassanese, Corolea blend
S_16	CAL_41	Calabria (Italy)	Nocellara del Belice monovarietal
S_17	SIC_90	Sicily (Italy)	Moresca, Nocellara del Belice, Frantoio blend
S_18	SIC_92	Sicily (Italy)	Blend
S_19	SIC_93	Sicily (Italy)	Tonda Iblea monovarietal
S_20	SIC_94	Sicily (Italy)	Blend
S_21	SIC_95	Sicily (Italy)	Tonda Iblea monovarietal
S_22	SIC_96	Sicily (Italy)	Tonda Iblea monovarietal
S_23	SIC_97	Sicily (Italy)	Tonda Iblea monovarietal
S_24	SIC_98	Sicily (Italy)	Tonda Iblea monovarietal
S_25	SIC_99	Sicily (Italy)	Tonda Iblea monovarietal
S_26	GRE_113	Greece	Koroneiki monovarietal
S_27	GRE_116	Greece	Blend
S_28	GRE_118	Greece	Kolovi monovarietal
S_29	GRE_120	Greece	n.p.
S_30	GRE_124	Greece	Koroneiki monovarietal
S_31	SIC_128	Sicily (Italy)	Blend: Minuta, Nocellara
S_32	SIC_129	Sicily (Italy)	Blend from Valdemone
S_33	SIC_130	Sicily (Italy)	Blend from Messina province

Table S2. Quantitative data and relative deviation for 20 main variables, as measured through MARA-NMR processing method working on mono dimensional ^{1}H and ^{13}C-NMR experiments for 33 samples.

Samples	TG%	1,2-DG%	1,3-DG%	SQmol%	Ln%	L%	O%	PO%	V%	P%	S%	Lni%	Li%	Oi%	POi%	Vi%	Pi%	Si%	VSTR	CYSR
S_1	96.7	1.2	2.1	1.7	0.6	10.2	63.9	1.3	2.8	19.1	2.2	n.d.	5.0	26.9	n.d.	1.4	n.d.	n.d.	4501	476
S_2	97.4	1.3	1.3	2.2	0.6	12.5	61.8	0.9	2.5	19.7	1.9	n.d.	7.6	24.7	0.7	0.1	0.1	0.1	2578	314
S_3	97.7	1.6	0.7	1.3	0.5	12.6	59.4	0.8	3.7	21.1	2.0	n.d.	8.5	21.6	n.d.	n.d.	1.3	2.0	3518	298
S_4	97.4	1.4	1.2	0.8	0.6	15.4	51.9	1.1	4.9	23.7	2.4	n.d.	8.9	21.1	n.d.	n.d.	0.9	2.4	4987	597
S_5	97.2	2.1	0.7	2.9	0.7	5.7	72.8	0.7	1.8	16.0	2.4	n.d.	2.9	29.4	0.7	0.3	n.d.	n.d.	2310	0
S_6	97.3	1.7	1.0	2.7	0.6	2.6	73.9	0.7	3.5	16.7	1.9	n.d.	0.6	31.9	0.7	n.d.	n.d.	0.1	1122	0
S_7	97.3	2.0	0.8	2.5	0.6	3.1	74.2	0.8	2.7	16.1	2.5	n.d.	2.0	29.9	0.8	n.d.	n.d.	0.7	1863	0
S_8	97.7	1.6	0.7	2.2	0.6	6.4	70.8	0.9	3.6	15.5	2.1	n.d.	3.3	29.0	n.d.	n.d.	n.d.	1.0	3477	533
S_9	96.8	1.8	1.4	1.3	0.4	10.2	6n.d.	1.1	5.0	21.7	1.6	0.4	6.0	23.7	n.d.	0.9	2.3	n.d.	2300	0
S_10	96.9	1.3	1.8	2.3	0.7	7.6	65.2	1.1	3.4	2n.d.	2.1	n.d.	5.5	26.2	n.d.	0.8	0.9	n.d.	2742	544
S_11	97.4	1.2	1.4	2.9	0.6	5.9	67.2	1.2	3.6	19.2	2.4	0.6	4.1	26.9	n.d.	n.d.	n.d.	1.7	4009	1083
S_12	97.6	1.3	1.0	3.1	0.6	7.7	65.4	2.5	4.6	17.4	1.9	n.d.	5.8	27.5	n.d.	n.d.	n.d.	n.d.	4153	1041
S_13	97.7	1.5	0.9	2.8	0.8	8.3	67.1	1.0	2.4	18.3	2.0	n.d.	3.1	29.2	1.0	n.d.	n.d.	n.d.	1923	0
S_14	97.8	1.4	0.8	3.4	0.6	4.5	71.3	1.2	2.5	17.7	2.3	n.d.	4.3	27.9	1.2	n.d.	n.d.	n.d.	2311	31
S_15	97.8	1.1	1.1	4.0	0.6	8.3	64.0	0.7	3.1	20.7	2.7	n.d.	5.3	27.3	0.7	n.d.	n.d.	n.d.	2848	533
S_16	97.8	1.5	0.7	3.1	0.6	6.2	70.2	0.6	3.0	17.5	2.0	n.d.	3.0	28.7	n.d.	0.9	0.1	0.6	1479	3
S_17	97.4	1.6	1.0	2.1	0.6	6.4	68.5	1.0	3.3	18.1	2.0	0.5	4.1	27.2	0.6	0.2	n.d.	0.8	2943	376
S_18	97.6	1.1	1.3	2.2	0.5	6.7	68.8	1.1	2.4	18.5	2.0	n.d.	2.5	29.6	0.9	0.1	0.1	0.1	3115	586
S_19	97.6	1.4	1.0	3.5	0.8	7.5	65.4	1.0	2.9	20.3	2.1	0.1	3.7	27.1	0.5	n.d.	0.8	1.0	3756	374

S_20	97.2	1.6	1.2	2.0	0.6	9.3	64.8	1.1	3.7	19.0	1.5	n.d.	6.2	24.9	n.d.	0.6	1.6	n.d.	2788	242
S_21	97.2	1.7	1.1	3.0	0.7	7.6	66.2	0.6	3.1	19.9	1.9	n.d.	2.9	29.8	0.6	n.d.	n.d.	n.d.	3313	220
S_22	97.7	1.5	0.8	3.6	0.7	7.5	64.8	1.3	3.4	20.7	1.7	n.d.	3.8	28.2	n.d.	n.d.	1.4	n.d.	3031	238
S_23	98.0	1.6	0.4	3.4	0.8	6.9	65.4	1.4	2.8	20.1	2.5	n.d.	3.8	27.3	n.d.	0.1	n.d.	2.1	2830	0
S_24	97.6	1.6	0.8	3.4	0.7	7.9	65.9	1.1	2.5	2n.d.	2.0	0.7	4.5	27.4	n.d.	n.d.	0.7	n.d.	2622	51
S_25	98.0	1.2	0.8	2.4	0.6	7.2	66.9	0.6	3.6	19.2	2.0	n.d.	4.0	27.7	n.d.	n.d.	n.d.	1.5	3967	761
S_26	97.3	1.3	1.4	2.0	0.7	4.4	73.3	1.3	3.0	15.6	1.8	n.d.	0.6	32.7	n.d.	n.d.	n.d.	n.d.	1620	36
S_27	97.4	1.5	1.1	2.5	0.6	4.4	72.2	0.9	2.6	16.9	2.3	0.6	4.3	26.6	n.d.	n.d.	1.8	n.d.	2883	542
S_28	97.8	1.3	0.9	2.5	0.6	7.1	73.9	1.3	1.5	13.7	1.8	0.6	4.5	27.3	n.d.	n.d.	0.8	n.d.	3444	371
S_29	96.9	1.4	1.6	2.7	0.7	5.2	70.5	1.4	2.6	17.1	2.4	n.d.	4.0	26.9	n.d.	0.6	n.d.	1.8	3054	555
S_30	97.9	1.2	0.9	1.9	0.6	4.8	73.1	0.9	2.9	15.4	2.3	0.6	2.1	28.8	0.9	n.d.	n.d.	0.9	2899	428
S_31	96.9	2.5	0.5	2.6	0.6	6.3	64.5	0.5	5.2	21.5	1.4	n.d.	3.6	28.7	n.d.	n.d.	1.0	n.d.	2695	247
S_32	96.0	3.0	1.0	3.1	0.7	9.4	61.8	1.0	4.4	21.1	1.8	0.7	4.7	26.1	0.6	0.6	0.1	0.6	2681	168
S_33	95.8	2.3	1.9	2.2	0.7	8.7	65.6	1.0	3.9	18.3	1.9	0.7	4.9	26.9	n.d.	n.d.	0.3	0.6	3136	323

Symbols of compounds are explained in the Table 1 in the main text. We point out the great potential of the MARA NMR also estimating the internal/external position of the fatty esters.

4. Discussion

5. Conclusions

This study definitely assesses the constant and relevant presence in olive oils of a not-oleic mono-unsaturated fatty ester called cis-vaccenic ester. It resolves the literature controversies concerning the assignment of some ^{13}C-NMR resonances but, more importantly, it brings back the expected coherency between NMR and chromatography data. The serendipitos comparison of GC-FID and NMR profiles for the "exotic" Capparis Spinosa oil evidenced the overwhelming amount of cis-vaccenic ester in this matrix but also unambiguously confirmed ^{13}C-NMR assignments also validated in olive oil. By reconsidering the NMR and GC-FID of olive oils, it turned out the surprising quantitative contribution (around 3%) of cis-vaccenic ester. The official GC method does not always performs an acceptable resolution to resolve and quantify oleic and cis-vaccenic esters and thi s is leading to the undistinguished quantification of both mono-unsaturated fatty esters. It opens up a great potential for any technique able to clearly resolve cis-vaccenic moyeties (just like 13C-NMR) in the study of extra-virgin olive oils.

6. Patents

This section is not mandatory, but may be added if there are patents resulting from the work reported in this manuscript.

Appendix A

MARA-NMR

Table S3. General scheme of MARA-NMR referred just to the first sample. There are several blocks: namely ^{1}H-NMR integrations with assignment (100 entries), DPFGSE ^{1}H-NMR integrations (17 entries, **not used in this study**), ^{13}C-NMR integrations (90 entries) along with some sum of integrals belonging to the same spectral block. Where possible, assignments are performed respecting the chemical position indicated also in other studies about the NMR of olive oil compounds (see Figure 1), for the fatty esters the abbreviation is followed by a number indicating the distance from the carboxyl position (generally from 1 to 18). These first rows will be used in the following equations according to the style of (1) (see main text) conveyed as square sum in the raw called RHO. The RHO value is minimized playing around with the quantitative variables so that the theoretical outcome is best-fitting the real (independent) variables.

**Groups**	**Assignments**	**Integrat Ion Label**	**Integral Range**		
	Chemical label				S_1
	Saturated linear aldehydes	I_1	9.77	9.746	0.064
	5S,4R(TY-EA-DA+HTY-EA-DA)	I_2	9.694	9.674	0.029
	Aldehyde A	I_3	9.674	9.659	0.016
	HTY-EDA-DA (Oleocanthal 3-C-H)	I_4	9.65	9.634	0.025
	TY-EDA-DA (Olacein 3-C-H)	I_5	9.634	9.622	0.000
	HTY-EA-CYA	I_6	9.534	9.519	0.025

	TY-EA-CYA+1/2(2E-hexenal)+1/2(2E-alkenal)	I_7	9.519	9.505	0.012
	1/2(2E-hexenal)	I_8	9.505	9.496	0.004
	1/2(2E-alkenal)	I_9	9.496	9.491	0.004
	5S,4S(TY-EA-DA+HTY-EA-DA)	I_10	9.465	9.446	0.011
	Aldehyde B	I_11	9.364	9.351	0.018
	Aldehyde C	I_12	9.328	9.309	0.008
	Aldehyde D	I_13	9.289	9.28	0.008
	ELENOLIDE see magiatis et al 2019	I_14	9.28	9.264	0.005
	TY-EDA-DA (Olacein 1-C-H)	I_15	9.24	9.228	0.014
	HTY-EDA-DA (Oleocanthal 1-C-H)+TY-EA-DA+TY-EA-EN	I_16	9.228	9.211	0.003
	HTY-EA-DA+HTY-EA-EN	I_17	9.211	9.19	0.005
	Unidentified water exchanging protons	I_18	8.302	8.106	0.449
	unknown singlet	I_19	8.106	8.09	0.019
	unidentified hydroxyl groups	I_20	8.09	8.007	0.062
	formaldehyde	I_21	8.007	7.996	0.027
	phtalate A	I_22	7.728	7.694	0.032
	Polyphenol A	I_23	7.683	7.66	0.004
	ELENOLIDE-3-CH	I_24	7.65	7.64	0.000
	unknown	I_25	7.594	7.58	0.008
	TY-EA-CYH (3-CH)	I_26	7.58	7.562	0.008
	HTY-EA-CYH (3-CH)	I_27	7.562	7.547	0.004
	phtalate B	I_28	7.538	7.502	0.038
	CDCl3 sat A	I_29	7.5	7.47	0.058
	half 1 TY-EA-CYA	I_30	7.413	7.404	0.003
	half 1 HTY-EA-CYA	I_31	7.404	7.397	0.003
	half 2 TY-EA-CYA	I_32	7.386	7.378	0.007
	half 2 HTY-EA-CYA	I_33	7.378	7.371	0.008
	unknown cycloene-eter A	I_34	7.354	7.347	0.017

	unknown cycloene -eter B	I_35	7.347	7.338	0.029
	CDCl3	I_36	7.302	7.226	8.519
	SatB CDCl3+ TY-Derivates (4-CH, 2H)	I_37	7.1	7	0.110
	unknown polyphenols B	I_38	6.907	6.814	0.053
	TY-derivates (7-CH, 2H)+ HTY-derivates (7,8-CH, 2H)	I_39	6.814	6.667	0.125
	HTY-Derivates (4-CH, 1H)+ (Z,E)peroxideA+ ELENOLIDE-8-CH	I_40	6.667	6.507	0.722
	unknown polyphenols	I_41	6.488	6.438	0.115
	(E,E)peroxide A	I_42	6.326	6.209	0.286
	(E,E)peroxide B	I_43	6.186	6.058	0.222
	(Z,E)peroxide B	I_44	6.04	5.961	0.542
	unknown	I_45	5.961	5.837	0.202
	(E,E)peroxide C	I_46	5.802	5.692	0.519
	unknown	I_47	5.693	5.612	0.431
	(Z,E)peroxide C	I_48	5.611	5.555	0.701
	(Z,E)peroxide D	I_49	5.556	5.532	0.458
	alkenes+(E,E)peroxide D	I_50	5.532	5.44	5.650
	CH=CH	I_51	5.44	5.29	857.702
	CH-TG	I_52	5.29	5.223	158.600
	(8CH)SQ+1,2-DG	I_53	5.223	5.051	12.305
	terpeneXX	I_54	5.051	4.836	0.539
	terpeneX+residual water	I_55	4.759	4.723	0.162
	terpeneC	I_56	4.723	4.695	0.570
	Group C=CH2 of sterols: Gramisterol/Obtusifoliol/Cycloleucalenol/ 24-Methylenecycloartanol	I_57	4.674	4.642	0.409
	Geranylgeraniol and phytol esters (GERG and PHYT)	I_58	4.605	4.564	0.487
	satB-CH2''-TG	I_59	4.479	4.409	1.597
	CH2''-TG+2(1,2-DG)	I_60	4.365	4.204	327.739
	CH2'-TG+5(1,3-DG)	I_61	4.204	4.049	340.910

acetate-esters+ satellite CH2' overlap	I_62	4.049	4.003	3.032
satA-CH2'-TG partial	I_63	4.003	3.954	1.014
	I_64	3.826	3.768	0.586
1,2-DG	I_65	3.769	3.662	3.966
unknown	I_66	3.662	3.561	1.404
(◎-sitosterol + ◎5-avenasterol + ◎5-campesterol) >**CH**-OH	I_67	3.561	3.474	1.261
unknown singlet	I_68	3.474	3.464	0.112
	I_69	3.464	3.422	0.425
	I_70	3.422	3.359	0.618
Cycloartenol+ 24-Methylenecycloartanol (3->C**H**-OH)	I_71	3.304	3.246	0.951
Cicloeucalenol+ gramisterol (>C**H**-OH)	I_72	3.162	3.069	1.138
maslinic acid+ urosolic acid	I_73	3.052	3.006	0.639
satB-divinylCH2tufa shoulder	I_74	2.968	2.92	0.918
satB-divinylCH2	I_75	2.921	2.868	1.634
divynilCH2-TUFA	I_76	2.854	2.798	8.810
divynilCH2-DUFA	I_77	2.798	2.732	104.636
foot of the divynil signal	I_78	2.732	2.689	1.501
satA-divinylCH2+X	I_79	2.67	2.555	2.868
satB-a-CH2	I_80	2.477	2.402	8.731
◎-CH2	I_81	2.364	2.25	1000.000
satb-vinylCH2+satA-a-CH2	I_82	2.217	2.112	20.435
vinylCH2-pufa+20(H) vinyl CH2-SQ	I_83	2.109	1.937	1630.670
satA-vynilCH2	I_84	1.921	1.838	17.490
interspace	I_85	1.838	1.779	6.554
residual water	I_86	1.778	1.696	14.111
1-SQ-CH3-6H*(SQ)	I_87	1.696	1.665	16.372

	◎-CH2+18H*(SQ)	I_88	1.665	1.521	1033.270
	satb-CH2-FA	I_89	1.521	1.402	71.079
	CH2-FA	I_90	1.402	1.193	9574.030
	sat CH2-FA	I_91	1.194	1.065	91.374
	3/4satB-FA	I_92	1.041	0.995	9.523
	Me-◎3-FA+satB/4	I_93	0.995	0.95	10.883
	Me-FA	I_94	0.95	0.825	1337.720
	satA-FA	I_95	0.774	0.727	7.404
	(b-sitosterol + D5-avenasterol + D5-campesterol) 18-CH_3	I_96	0.694	0.671	2.250
	Gramisterol*3H+Cycloartenol+ 24-Methylenecycloartanol (endo, 19CH_2)	I_97	0.576	0.548	0.238
	other sterol	I_98	0.547	0.519	0.560
	Cycloartenol+ 24-Methylenecycloartanol (exo, 19CH_2	I_99	0.354	0.314	0.514
	TMS	I_100	0.02	-0.02	34.219
					S_1
	Assignments	Integration_Label	Integral Range	0.000	
	Saturated linear aldehydes	I_D_1	9.77	9.746	49.622
	5S,4R(TY-EA-DA+HTY-EA-DA)	I_D_2	9.694	9.674	10.109
	Aldehyde A	I_D_3	9.674	9.659	40.656
	HTY-EDA-DA (Oleocanthal 3-C-H)	I_D_4	9.65	9.634	85.794
	TY-EDA-DA (Olacein 3-C-H)	I_D_5	9.634	9.622	100.000
	HTY-EA-CYA	I_D_6	9.534	9.519	18.810
	TY-EA-CYA+1/2(2E-hexenal)+1/2(2E-alkenal)	I_D_7	9.519	9.505	23.111
	1/2(2E-hexenal)	I_D_8	9.505	9.496	8.377
	1/2(2E-alkenal)	I_D_9	9.496	9.491	1.749
	5S,4S(TY-EA-DA+HTY-EA-DA)	I_D_10	9.465	9.446	−2.631

	Aldehyde B	I_D_11	9.364	9.351	3.743
	Aldehyde C	I_D_12	9.328	9.309	6.574
	Aldehyde D	I_D_13	9.289	9.28	9.610
	ELENOLIDE	I_D_14	9.28	9.264	8.560
	TY-EDA-DA (Olacein 1-C-H)	I_D_15	9.24	9.228	85.664
	HTY-EDA-DA (Oleocanthal 1-C-H)+TY-EA-DA+TY-EA-EN	I_D_16	9.228	9.211	73.169
	HTY-EA-DA+HTY-EA-EN	I_D_17	9.211	9.19	10.275
	Assignments	Integration_Label	Integral Range		
					S_1
	Pext1+Sext1	I_13C_1	173.242	173.21	9.693
	Oext1+Lext1+POext1+Lnext1+Vext1	I_13C_2	173.21	173.172	24.661
	Oint1+Lint1+Pint1+Sint1+POint1+Eint1	I_13C_3	172.808	172.768	17.673
	SQ10(q)	I_13C_4	135.084	135.015	0.500
	SQ6(q)	I_13C_5	134.896	134.793	1.090
	Ln16	I_13C_6	131.959	131.892	0.842
	SQ2(q)	I_13C_7	131.193	131.116	1.337
CH toward the tail of fatty ester L13+Ln9+O10+PO10+L9+V12= 2Ln+2L+O+PO+V	L13+Ln9	I_13C_8	130.224	130.157	13.622
	Oint10	I_13C_9	130.037	130.014	29.662
	Oext10+POint10	I_13C_10	130.014	129.989	43.055
	Lext9+POext10	I_13C_11	129.989	129.97	8.554
	Lint9	I_13C_12	129.97	129.944	6.261
	V12	I_13C_13	129.935	129.91	2.848
CH toward the head of fatty ester V11+O9+PO9+Ln12+Ln13+L10+L12= 2Ln+2L+PO+O+V	V11	I_13C_14	129.839	129.818	3.050
	Oext9	I_13C_15	129.729	129.696	40.723
	Oint9+PO9	I_13C_16	129.696	129.666	28.969
	Ln12	I_13C_17	128.323	128.283	0.644
	Ln13	I_13C_18	128.265	128.217	0.769

	L10	I_13C_19	128.129	128.075	12.563
	L12	I_13C_20	127.956	127.898	10.504
	Ln10	I_13C_21	127.815	127.761	0.771
	Ln15	I_13C_22	127.17	127.116	0.147
	SQ3	I_13C_23	124.472	124.441	1.084
	SQ11	I_13C_24	124.369	124.332	0.805
	SQ7	I_13C_25	124.332	124.301	1.097
	CHOH	I_13C_26	68.974	68.894	32.623
	CH2OH	I_13C_27	62.188	62.034	68.405
	SQ9	I_13C_28	39.795	39.775	1.211
	SQ5	I_13C_29	39.775	39.753	0.927
(P+S+O+E+PO+L+Ln)2	(Pint+Sint+Oint+Vint+POint+Lint+Lnint)2	I_13C_30	34.233	34.185	33.333
	Pext2+Sext2+Vext2	I_13C_31	34.082	34.053	21.121
	Oext2+Lext2+Lnext2	I_13C_32	34.053	34.025	46.016
E18+(S+O+E+L)16+(PO+P)14+(UNK)= E+PO+P+S+O+E+L	P14+S16	I_13C_33	31.982	31.959	21.649
	O16	I_13C_34	31.959	31.928	67.470
	V16	I_13C_35	31.839	31.817	3.034
	PO14	I_13C_36	31.817	31.8	1.395
	L16	I_13C_37	31.574	31.55	11.106
PO13+O12+V14+S14+S13+S12+S11+O7+S10+P12+P11+P10+PO12 =2O+V+5S+3P	PO13	I_13C_38	29.947	29.919	0.758
	O12	I_13C_39	29.827	29.784	78.794
	V14+V13	I_13C_40	29.784	29.762	9.320
	S14+S13+S12+S11+O7+S10+P12+P11+P10+PO12=5S+3P+O+PO	I_13C_41	29.762	29.712	151.653
P7+L7+Ln7+P8+P9+S7+S5+P5+V7+V	Pint7+PO7+P8+P9+S7+S8+S9	I_13C_42	29.712	29.686	44.125

15+O14+S8+S9=4P +4S+L+O+2V	Pext7+Lint7	I_13C_43	29.677	29.649	29.668
	Lext7+V7ext	I_13C_44	29.649	29.634	9.178
	not assigned	I_13C_45	29.634	29.618	4.189
	O14+Sint5	I_13C_46	29.591	29.547	75.118
	Sext5+Pint5	I_13C_47	29.547	29.53	4.409
	Pext5	I_13C_48	29.53	29.5	21.200
	Ve7	I_13C_49	29.5	29.486	3.326
	Vi7	I_13C_50	29.486	29.463	3.793
S15+P13+L15+O15 +O13+Si6+Vi5+P6 +Se6+Ve5 = 2S+2P+2O+V+L	S15	I_13C_51	29.436	29.416	3.857
	P13	I_13C_52	29.416	29.393	22.638
	L15	I_13C_53	29.393	29.379	15.313
	O13+O15+V15+Sint6	I_13C_54	29.379	29.339	149.649
	P6+V5+Sext6	I_13C_55	29.335	29.279	30.687
O5+O6+O4+V6+L 5+L4+L6+Ln4+Ln5 +Ln6+P4+S4+V4+ PO6+PO5+PO4= 3O+3L+3Ln+2V+3 PO+P+S	Oint5	I_13C_56	29.249	29.219	38.575
	Oext5+Lint5+Lnint5+ Lext5+Lext5+POint5= Oext+L+Ln+Point	I_13C_57	29.22	29.182	53.523
	(Oext6+Vext6+Lint6+ Lnint6)+(Pext4+Sext4 +POext5+Oint6+Vint 6)= O+(L+Ln+E)int+(S+P+ PO)ext	I_13C_58	29.182	29.13	115.641
	Oext4+Pint4+Sint4+L ext6+Lnext6+POint6	I_13C_59	29.13	29.098	52.729
	Oint4+Lext4+Lnext4+ P4+ Oext6+POext4	I_13C_60	29.098	29.06	36.298
	POint4+Lint4+Lnint4 +V4	I_13C_61	29.04	29.005	5.447
	SQ12	I_13C_62	28.313	28.292	1.198
O8+PO8+L8+L14+ Ln8+V9+PO11+O1 1+V13=2O+2V+P O+2L+Ln	O11+V13	I_13C_63	27.275	27.242	77.957
	L14+V9+Ln8+PO11	I_13C_64	27.242	27.226	20.192

	L8	I_13C_65	27.226	27.214	14.576
	O8	I_13C_66	27.214	27.176	75.479
	SQ4	I_13C_67	26.819	26.794	1.161
	SQ8	I_13C_68	26.7	26.672	1.502
	SQ1	I_13C_69	25.708	25.688	1.055
	L11	I_13C_70	25.672	25.651	12.208
	Ln11	I_13C_71	25.651	25.637	1.655
	Ln14	I_13C_72	25.567	25.548	0.594
(O+L+P+S+E+PO+Ln)3	O3i+P3i+S3i	I_13C_73	24.938	24.907	28.433
	Li3+Lni3+Pe3+Se3+Vi3	I_13C_74	24.907	24.889	29.767
	Oe3+Le3+Ve3+Lne3+PO3	I_13C_75	24.889	24.855	50.392
O17+E19+L17+P15+S17+Ln17	P15+S17	I_13C_76	22.737	22.722	21.361
	O17	I_13C_77	22.722	22.699	60.480
	V17	I_13C_78	22.699	22.685	5.293
	L17	I_13C_79	22.625	22.595	10.927
	PO15	I_13C_80	22.58	22.558	1.295
	Ln17	I_13C_81	20.601	20.545	0.987
	SQ13	I_13C_82	17.701	17.644	1.382
	SQ14	I_13C_83	16.063	16.024	0.973
	SQ15	I_13C_84	16.024	15.984	1.238
O18+P16+S18+E20+L18+PO16	Ln18	I_13C_85	14.306	14.274	1.277
	unk	I_13C_86	14.274	14.24	0.741
	O18+V18+P16+S18	I_13C_87	14.162	14.102	84.676
	L18+PO16	I_13C_88	14.102	14.072	12.078
	UNK	I_13C_89	13.997	13.964	0.426

	TMS	I_13C_90	0.032	-0.03	1.695
0	Summ Block C-1				52.027
0	Summ aromatic C10=2Ln+2L+O+PO+V				104.844
0	Summ aromatic C9=2Ln+2L+O+PO+V				97.222
0	Summ a-CH2				100.470
0	Summ CH2-omega3				106.309
	CH2- block I				240.526
	CH2- block II				190.818
	CH2- block III				222.144
	CH2- block IV				302.214
	Vynil-CH2 tot				188.204
0	Summ b-CH_2				108.592
0	Summ omega2-CH2				100.343
0	Summ of terminal Me groups				98.031
	DPFGSE_equations				
					S_1
	5S,4R(TY-EA-DA+HTY-EA-DA)				10.109
	HTY-EDA-DA (Olacein 3-C-H)				35.780
	TY-EDA-DA (Oleocanthal 3-C-H)				31.317
	5S,4S(TY-EA-DA+HTY-EA-DA)				-2.631
	TY-EDA-DA (Oleocanthal 1-C-H)				16.981
	HTY-EDA-DA (Olacein 1-C-H)+TY-EA-DA+TY-EA-EN				14.695
	HTY-EA-DA+HTY-EA-EN				10.275
	DPFGSE summ tot_rho				2979.906

		S_1
	sat_lin_ald_ppm_mol	297.734
Ligstroside aglycone	TY-EA-CYA	77.906
Oleuropein aglycone	HTY-EA-CYA	112.859
	2E-hexenal	50.265
	other 2E-alkenals	10.496
	optimized	
	HTY-EDA-DA (Olacein)	300.080
	TY-EDA-DA (Oleocanthal)	412.101
	5S,4R-TY-EA-DA	0.000
	5S,4S-TY-EA-DA	0.000
	TY-EA-EN	50.765
	5S,4R-HTY-EA-DA	0.000
	5S,4S-HTY-EA-DA	0.000
	HTY-EA-EN	0.000
	TY-EA	729.843
	HTY-EA	112.859
		TUR_5
	Sample	S_1
	◎-SIT+avenaster+camposter	4500.740
	Cyclo-sterols	476.116
		0.000
	Sample	0.000
	Average FW	870.621
	TG%	96.687
	1,2-DG%	1.189
	1,3-DG%	2.124
	Sqmol%	1.675
	Ln%	0.587
	L%	10.178
	O%	63.073
	PO%	2.093
	V%	2.813
	P%	19.000
	S%	2.256

				0.000
				0.000
				0.000
	Ln(int)%			0.001
	L(int)%			5.183
	O(int)%			26.575
	PO(int)%			0.000
	V(int)%			1.574
	P(int)%			0.000
	S(int)%			0.000
				0.000
				0.000
				pp
				TUR_5
eq_H_1	aliphatic-CH2			−0.388
eq_H_2	SQ_terminal			0.741
eq_H_3	SQ+◎CH2			−0.880
eq_H_4	Ln - omega3-uns			0.083
eq_H_5	vynilCH2tot			−0.057
eq_H_6	divynilCH2-dufa			−0.162
eq_H_7	divynilCH2-tufa			−0.005
eq_H_8	CH=CH			0.709
eq_H_9	1,2-DG%			0.011
eq_H_10	TG+5(1,3-DG)			0.184
eq_H_11	2*TG+2(1,2-DG)			0.297
				S_1
satqExt	S1+P1qExt	I_13C_1	eq_13C_1	−0.075
SQ(Cq)	SQ(Cq)	I_13C_4/5	eq_13C_2	0.011
Oint/Oext	Oint/Oext	I_13C_9/15	eq_13C_3	−0.023
Ln16			eq_13C_4	0.029
(Ln16)+L13+Ln9+Oint10+Oext10+POint10+Lext9+POext10+Lint9 = L13+L9+PO10+O10+Ln9 = 2Ln+2L+O+PO	L13+Ln9	I_13C_8	eq_13C_5	0.193
	Oint10	I_13C_9	eq_13C_6	-0.140
	Oext10+POint10	I_13C_10	eq_13C_7	0.029
	Lext9+POext10	I_13C_11	eq_13C_8	0.024
	Lint9	I_13C_12	eq_13C_9	0.036

V12	V	I_13C_13	eq_13C_10	−0.255
CH toward the head of fatty ester O9+PO9+Ln12+Ln13+L10+L12+V11=O+PO+2Ln+2L+V	V11	I_13C_14	eq_13C_11	−0.003
	Oext9	I_13C_15	eq_13C_12	0.222
	Oint9+PO9	I_13C_16	eq_13C_13	−0.223
	Ln%(12,13-CH)	I_13C_17	eq_13C_14	0.028
	L%(CH)	I_13C_18	eq_13C_15	0.394
	Ln%(10,15-CH)	I_13C_19	eq_13C_16	−0.004
SQ(CH)	SQ(CH)	I_13C_15	eq_13C_17	−0.101
SQ(9-5-CH2)	SQ(9-5-CH2)	I_13C_16	eq_13C_18	−0.006
Pext2+Sext2+Vext2	Pext2+Sext2+Vext2/Oe2+Le2+Lne2	I_13C_31/32	eq_13C_19	−0.251
P14+S16+O16+V16+L16	P14+S16	I_13C_20	eq_13C_20	−0.446
	O16	I_13C_21	eq_13C_21	0.314
	V16	I_13C_22	eq_13C_22	0.024
	PO14	I_13C_23	eq_13C_23	−0.468
	L16	I_13C_24	eq_13C_24	0.162
PO13+O12+E14+S14+S13+S12+S11+O7+S10+P12+P11+P10+PO12 = 2O+V+2PO+5S+3P	PO13%	I_13C_38	eq_13C_25	−0.426
	O12	I_13C_39	eq_13C_26	0.938
	V14+V13		eq_13C_27	0.112
	S14+S13+S12+S11+O7+S10+P12+P11+P10+PO12=5S+3P+O+PO	I_13C_41	eq_13C_28	−0.126
Pint7+PO7+P8+P9+S7+S8+S9+Pext7+Lint7+Lext7+O14+Sint5+Sext5+Pint5+Pext5 = P7+P8+P9+S7+S8+S9+S5+P5+L7+O14+PO7 = 4(S+P)+O+L+PO	Pint7+PO7+P8+P9+S7+S8+S9= 2P+Pi+3S	I_13C_42	eq_13C_29	−0.419
	Pext7+Lint7	I_13C_43	eq_13C_30	0.159
	Lext7	I_13C_44	eq_13C_31	0.352
	O14+Sint5	I_13C_46	eq_13C_32	0.088
	Sext5+Pint5	I_13C_47	eq_13C_33	0.028

	Pext5	I_13C_48	eq_13C_34	−0.145
S15+P13+L15 = S+P+L	S15	I_C_51	eq_13C_35	0.411
	P13	I_C_52	eq_13C_36	−0.066
	L15	I_C_53	eq_13C_37	0.825
O13+O15+Sint6+E15+P6+E5+E6+Sext6 = 2O+V+P+S	O13+O15+Si5	I_13C_54	eq_13C_38	−0.187
	P6+V15+Se5	I_13C_55	eq_13C_39	0.162
Oint5+Oext5+L5+Ln5+POint5+O+(L+Ln+E)int5+(S+P+PO)ext5+Oext4+Pint4+Sint4+Eint4+Lext6+Lnext6+POint6+Oint4+Lext4+POext4+Lnext4+POext6POint4+Lint4+Lnint4 = 3Ln+3L+3O+3PO+E+P+S	Oint5+Li+Lni	I_13C_56	eq_13C_40	0.147
	Oext5+L5+Ln5+POint5	I_13C_57	eq_13C_41	0.042
	O+(L+Ln+E)int+(S+P+PO)ext	I_13C_58	eq_13C_42	0.021
	Oe4+Pi4+Si4+Ei4+Le6+Lne6+Poi6=(O+L+Ln)ext+(P+S+E+PO)int	I_13C_59	eq_13C_43	0.623
	Oint4+Lext4+POext4+Lnext4+POext6	I_13C_60	eq_13C_44	−0.287
	POint4+Lint4+Lnint4+Vint4	I_13C_42	eq_13C_45	−0.170
O8+V13+L14+V10+Ln8+PO11+L8+PO8+O11=2O+2E+2PO+2L+Ln	O11+V13	I_13C_63	eq_13C_46	−0.114
	L14+V10+Ln8+Po11	I_13C_64	eq_13C_47	0.148
	L8	I_13C_65	eq_13C_48	1.258
	O8	I_13C_66	eq_13C_49	−0.009
SQ(4-8-12-CH2)	SQ(4-8-12-CH2)	I_13C_48	eq_13C_50	0.020
L11%	L11%	I_13C_70	eq_13C_51	0.319
Ln11%	Ln11%	I_13C_71	eq_13C_52	0.187
Ln%	Ln14%	I_13C_72	eq_13C_53	−0.008
(O+P+S+E)int3+[(L+Ln)int+(P+S)ext]3+(O+L+E+Ln)ext3+PO3 = (Ln+L+O+PO+E+P+S) second methylene C atom	O3i+P3i+S3i=(O+P+S)int3	I_13C_51	eq_13C_54	−0.196
	Li3+Lni3+Pe3+Se3+Vi3=[(L+Ln)int+(P+S)ext]3	I_13C_52	eq_13C_55	−0.301
	Oe3+Le3+Ve3+Lne3+PO3=(O+L+E+Ln)e+PO	I_13C_53	eq_13C_56	0.497

(Ln+O+P+S+V+L+PO) last methylene carbon atom	P15+S17	I_13C_54	eq_13C_57	0.064
	O17	I_13C_55	eq_13C_58	−0.840
	V17	I_13C_56	eq_13C_59	0.985
	L17%	I_13C_57	eq_13C_60	0.569
	PO15	I_13C_58	eq_13C_61	−0.401
	Ln17	I_13C_59	eq_13C_62	0.032
SQ(Me-1,13,14,15)	SQ(Me-1,13,14,15)	I_13C_60	eq_13C_63	0.021
(Ln+O+P+S+E+L+PO) last carbon atom	Ln18%	I_13C_61	eq_13C_64	0.715
	Me(O+P+S+V)	I_13C_62	eq_13C_65	−0.765
	Me(L+PO)	I_13C_63	eq_13C_66	0.050
				TUR_5
				S_1
	⊚(dev)^2=rho			11.419
	Type ctrl+V to run minimization on the raw 387			11.419
	Constrain DG+TG			100.000
	Constrain total fatty acids			100.000
	Costrain internal position			33.333

© 2020 by the authors. Licensee MDPI, Basel, Switzerland. This article is an open access article distributed under the terms and conditions of the Creative Commons Attribution (CC BY) license (http://creativecommons.org/licenses/by/4.0/).
